# Predictors of glucocorticoid treatment intensity after endoscopic transsphenoidal surgery in patients with non-functional pituitary adenomas: a retrospective study

**DOI:** 10.3389/fneur.2026.1806623

**Published:** 2026-07-21

**Authors:** Mengzhen Yu, Chen Wang, Yukui Li, Jinli Sun, Zhangyu Li, Janyao Mao, Wanting Qi, Ping Zhong, Sifang Chen, Guowei Tan, Wentao Zheng, Xi Chen

**Affiliations:** 1 Department of Neurosurgery, The First Affiliated Hospital of Xiamen University, School of Medicine, Xiamen University, Xiamen, Fujian, China; 2 Department of Neurology, Xiamen Xianyue Hospital, Xianyue Hospital Affiliated with Xiamen Medical College, Fujian Psychiatric Center, Fujian Clinical Research Center for Mental Disorders, Xiamen, Fujian, China; 3 Department of Reproduction, The First Affiliated Hospital of Xiamen University, School of Medicine, Xiamen University, Xiamen, Fujian, China

**Keywords:** adrenal insufficiency, endoscopic transsphenoidal surgery, glucocorticoid replacement therapy, pituitary adenoma, postoperative hypopituitarism

## Abstract

**Background and objectives:**

Non-functioning pituitary adenomas (NFPAs) are among the most common pituitary tumors, accounting for approximately 22–54% of all pituitary adenomas. Although histologically benign, NFPAs can cause considerable morbidity and mortality due to their potential to induce endocrine disturbances. Endoscopic transsphenoidal surgery (eTSS) is the preferred treatment modality; however, postoperative hypopituitarism-particularly adrenal insufficiency-is frequent, necessitating long-term glucocorticoid therapy. Determining the optimal glucocorticoid dosage remains a significant clinical challenge. This study aimed to identify independent predictors of postoperative glucocorticoid therapy intensity in NFPA patients by integrating imaging and endocrinological parameters, and to develop a predictive model that supports individualized glucocorticoid replacement therapy and optimizes clinical decision-making.

**Methods:**

This retrospective analysis included 116 patients who underwent pituitary adenoma resection between March 2019 and December 2024. Demographic, clinical, radiological, endocrinological, and pathological data were collected. Imaging assessments included tumor volume, pituitary stalk stretch, and suprasellar extension measured using 3D Slicer software. Preoperative serum cortisol and ACTH levels were determined. Multivariate regression analysis was performed to identify independent predictors of postoperative glucocorticoid therapy intensity.

**Results:**

Tumor volume, suprasellar extension, pituitary stalk stretch, and preoperative morning cortisol levels were identified as independent predictors of glucocorticoid therapy intensity (all *p* < 0.05), collectively explaining 72.1% of the variance (adjusted *R*^2^ = 0.721). Bootstrap resampling confirmed model robustness (RMSE: 656.04; 95% CI: 637.50–690.22).

**Conclusion:**

Tumor volume, suprasellar extension, pituitary stalk stretch, and morning cortisol level are key determinants of postoperative glucocorticoid therapy intensity in NFPA patients. The proposed model provides an evidence-based tool to optimize postoperative care, potentially reducing the risk of adrenal insufficiency and overtreatment.

## Introduction

Non-functioning pituitary adenomas (NFPAs) are among the most prevalent types of pituitary tumors, accounting for approximately 22–54% of all pituitary adenomas (1–3). Unlike their functional counterparts, NFPAs do not secrete hormones and are typically characterized by symptoms related to local mass effect, including headaches, visual disturbances, and hypopituitarism (4, 5). Diagnosis is often incidental or made when patients present with unrelated symptoms. Despite their non-functional nature, these tumors can still result in substantial endocrine disturbances, particularly during the course of treatment.

Endoscopic transsphenoidal pituitary adenoma surgery (eTSS) has become one of the primary approaches for treating NFPAs due to its minimal invasiveness and enhanced visualization, which facilitate precise tumor resection while minimizing injury to surrounding structures (6). Nevertheless, despite the efficacy of surgical tumor removal, many NFPA patients develop postoperative hypopituitarism, most notably involving impairment of the adrenal cortical axis.

Hypopituitarism is a common postoperative complication in NFPA patients, especially when accompanied by adrenal insufficiency, which necessitates long-term glucocorticoid replacement therapy. However, postoperative glucocorticoid dosing is typically based on empirical judgment, lacking standardized quantitative guidelines. This creates a risk of inadequate replacement, which may increase the likelihood of adrenal crisis or lead to adverse reactions due to excessive replacement. Therefore, accurately assessing postoperative glucocorticoid requirements remains a critical issue in clinical practice (7, 8).

This study aims to identify factors predictive of postoperative glucocorticoid therapy intensity in NFPA patients and to develop a predictive model incorporating imaging and endocrinological markers. Such a model could enable individualized glucocorticoid replacement strategies, thereby optimizing clinical decision-making and reducing postoperative complications.

## Methods

### Study design

This retrospective study was approved by the Institutional Review Board (IRB) of the First Affiliated Hospital of Xiamen University and conducted in accordance with the principles of the Declaration of Helsinki (approval XMYY-2025KY182). We reviewed demographic, clinical, radiological, endocrinological, and pathological data from patients who underwent pituitary adenoma resection at our institution between March 2019 and December 2024.

The inclusion criteria were as follows, (1) radiological evidence of pituitary adenoma; (2) histopathological confirmation of non-functioning pituitary adenoma in accordance with the 2022 World Health Organization classification (9); (3) tumor resection via an endoscopic transnasal approach; (4) no prior history of surgery or radiotherapy; (5) availability of complete clinical data. Exclusion criteria included, (1) coexisting intracranial pathology; (2) functioning pituitary adenomas; (3) non-pituitary sellar lesions; (4) age < 18 years.

Because acute pituitary apoplexy may independently affect hypothalamic–pituitary–adrenal (HPA) axis function through sudden hemorrhagic or ischemic injury to the pituitary gland, inclusion of these patients could introduce substantial heterogeneity and confound the assessment of postoperative glucocorticoid requirements. Therefore, patients presenting with clinical and radiological evidence of acute pituitary apoplexy were excluded from the study cohort. Acute pituitary apoplexy was defined as the presence of acute clinical symptoms (e.g., sudden severe headache, visual deterioration, ophthalmoplegia, or altered consciousness) accompanied by radiological evidence of hemorrhagic or ischemic infarction within the pituitary adenoma.

**Figure 1:**
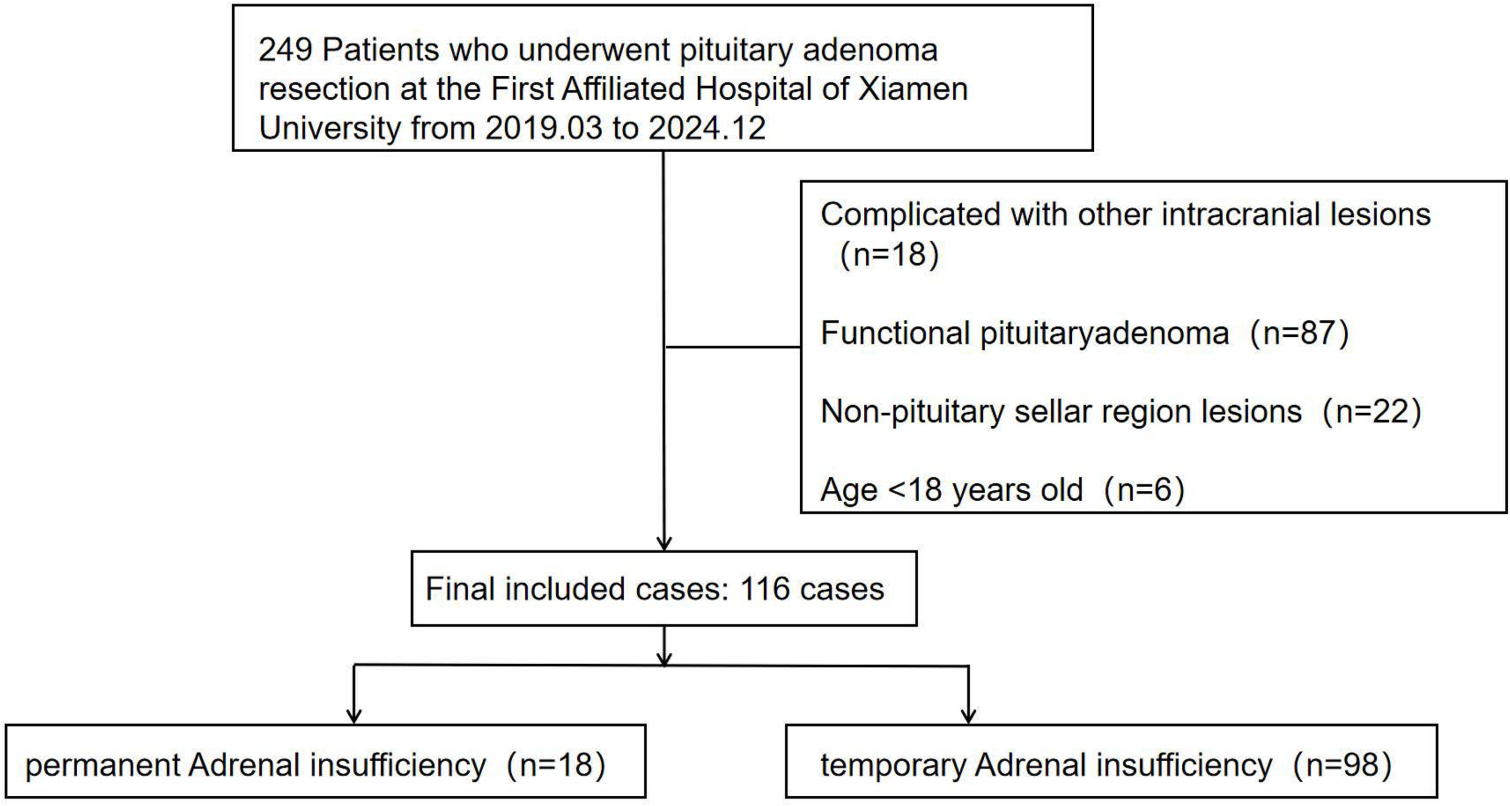
Flowchart of patients.

A total of 116 consecutive patients met the inclusion criteria. The study methodology and reporting followed the STROBE guidelines. Given the retrospective nature of this study, the IRB-consistent with national laws and institutional regulations-waived the requirement for informed consent. All patient-identifiable information was anonymized and replaced with coded identifiers (Figure 1).

### Radiological evaluation

#### Tumor volume

Imaging data of pituitary adenomas were exported using RadiAnt DICOM Viewer software and included preoperative and 3–6 months postoperative contrast-enhanced MRI scans as well as CT images. The data were saved in DICOM format and subsequently imported into 3D Slicer software for tumor reconstruction. The reconstruction process utilized the Volume Rendering, Segment Editor, Models, and General Registration modules, employing tools such as Paint, Grow from Seeds, and Threshold. Tumor volume was calculated precisely based on the reconstructed 3D model.

#### Tumor invasiveness

**Figure 2:**
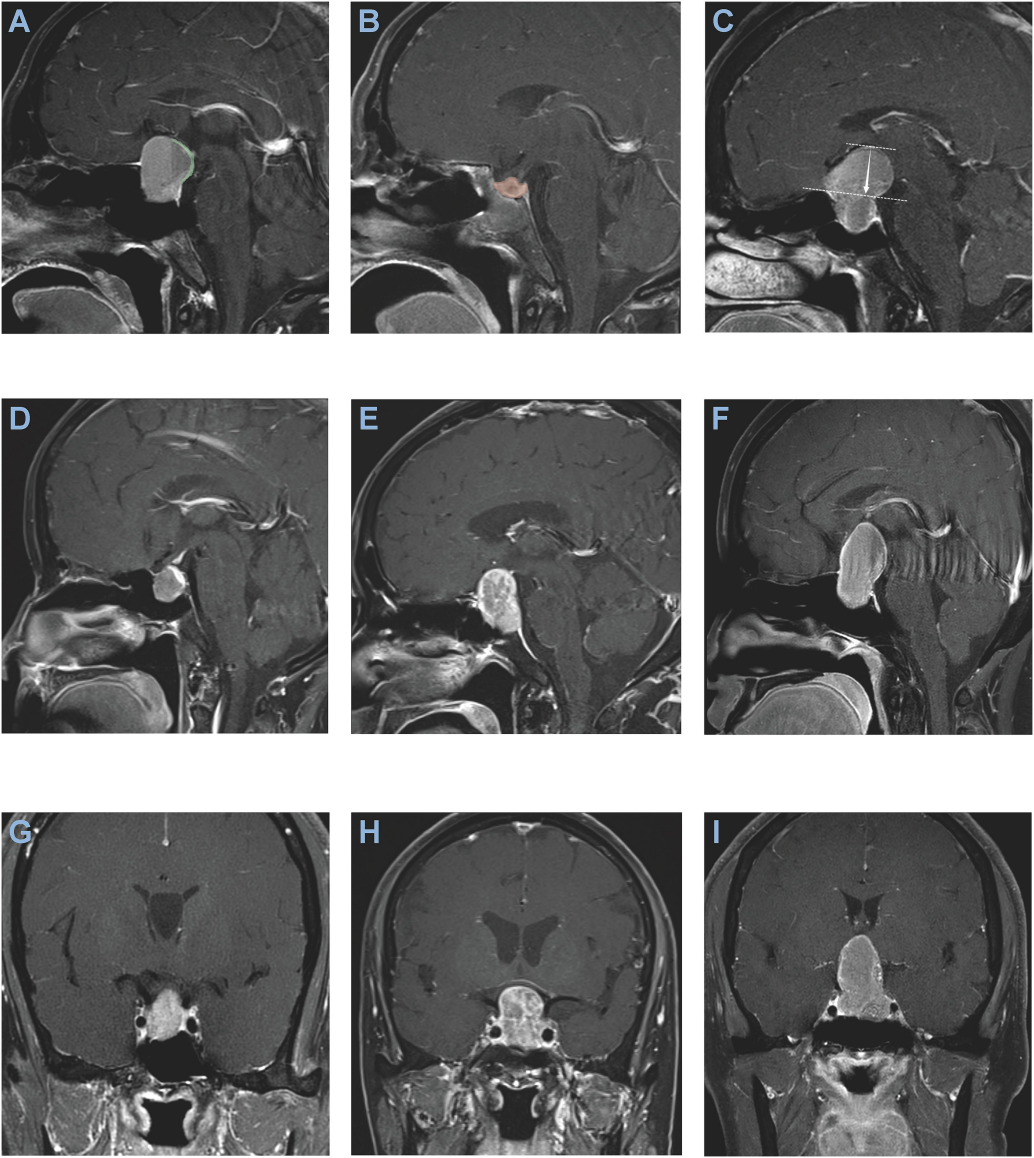
Radiological evaluation. **(A)** The green filled area indicates the preoperative location of the pituitary gland. **(B)** The red filled area indicates the postoperative pituitary position. **(C)** After aligning the MRI and CT images using 3D slicer software, the line connecting the anterior bed and the sella turcica (dashed line below) was obtained, and the vertical distance between the highest point of the tumor and this line was calculated. **(D)** Pituitary stalk type 1: no displacement. **(E)** Pituitary stalk type 2: displacement with discernible morphology. **(F)** Pituitary stalk type 3: indiscernible stalk. **(G)** Optic nerve type 1: no compression. **(H)** Optic nerve type 2: compression with recognizable deformation. **(I)** Optic nerve type 3: indiscernible optic nerve location.

Cavernous sinus invasion (CSI) was classified according to the Knosp grading system, with grades 3–4 considered indicative of CSI (10). Suprasellar extension was defined as the vertical distance from the tumor apex to the line connecting the anterior clinoid process and the dorsum sellae. After registering CT and MRI images using the General Registration module in 3D Slicer, the anterior clinoid process and dorsum sellae were identified on the CT scan, and the suprasellar extension was measured using the Markups module (Figure 2).

#### Pituitary stalk stretch

Preoperative and postoperative pituitary reconstructions were performed using 3D Slicer software. Due to morphological changes in the pituitary gland after surgery, calculating positional shifts of individual points was deemed imprecise. Therefore, we applied an affine transformation-based method to calculate overall displacement (11), with the displacement distance representing pituitary stalk stretch. A custom 3D Slicer module was developed for this purpose: preand postoperative MRI scans were registered, the pituitary model reconstructed, and the displacement distance determined by selecting corresponding anatomical points in the module (Figure 2).

#### Tumor texture

Tumor consistency was classified into “soft” or “hard” based on the signal intensity ratio (SIR = tumor signal intensity/non-enhanced normal white matter signal intensity) threshold of 1.92, as described by Romano et al. (12). Two regions of interest (ROIs) were manually placed on the three central slices from the first dynamic acquisition phase, avoiding non-solid components such as hemorrhage or cysts. A total of six ROIs were placed across all dynamic phases, and the mean SIR value was calculated. Each ROI was elliptical, with an area of 80 mm.

#### Pituitary stalk types and optic nerve types

Following the method of Hwang et al. (13) pituitary stalk morphology was categorized into three grades based on visibility (1) no displacement; (2) displacement with discernible morphology; and (3) indiscernible stalk (Figure 2). Optic nerve compression was also graded as (1) no compression; (2) compression with recognizable deformation; and (3) indiscernible optic nerve location (Figure 2).

#### Gross total resection (GTR) vs. non-gross total resection (non-GTR)

Extent of resection was assessed using contrast-enhanced MRI obtained approximately 3 months after surgery. GTR was defined as no radiological evidence of residual tumor, whereas non-GTR was defined as any detectable residual tumor on postoperative imaging.

### Endocrinological evaluation

Postoperative endocrine follow-up included assessment of the major pituitary axes, including the hypothalamic pituitary adrenal axis, hypothalamic pituitary thyroid axis, hypothalamic pituitary gonadal axis, and prolactin secretion. All hormonal measurements were routinely obtained at approximately 08:00 a.m. Hormonal function was reassessed every 2 weeks during follow-up. Clinical and biochemical evidence of diabetes insipidus was also recorded. In accordance with the 2016 Endocrine Society guidelines, adrenal insufficiency was defined based on early morning serum cortisol levels measured between 8:00 and 9:00 a.m.: values < 3 μg/dL indicated adrenal insufficiency; values > 15 μg/dL excluded it; and values between 3 and 15 μg/dL warranted an ACTH stimulation test, or the continued need for glucocorticoid replacement therapy based on clinical symptoms (14).

Based on previous studies of secondary adrenal insufficiency, it is generally accepted in clinical practice that failure of HPA axis function to recover within 1 year indicates permanent impairment. Accordingly, in the present study, patients who required glucocorticoid replacement after surgery and showed persistent adrenal insufficiency for ≥12 months were classified as having permanent AI, whereas all other cases were regarded as transient AI, and patients were consequently divided into these two groups (15).

#### Hormone replacement therapy protocol

For patients with confirmed or highly suspected AI diagnosed preoperatively, 100 mg intravenous hydrocortisone was administered during induction. Subsequently maintain via continuous infusion or intermittent injections to achieve a total dose of 200 mg/24 h until oral administration is feasible and transition to oral replacement dosage. For patients with normal or indeterminate preoperative HPA axis assessment, administer a single intravenous dose of 100 mg (or 50 mg) hydrocortisone during anesthesia induction. Administer as clinically indicated during the first 24–48 h postoperatively, and repeat morning cortisol testing at 08:00 on days 1–3 for initial screening. If postoperative cortisol levels are adequate, discontinue IV administration promptly and transition to oral therapy or cessation.

Postoperative maintenance dose: Oral prednisone is the preferred replacement medication, with a total daily dose generally ranging from 15 mg.

Monitoring During Replacement Therapy: Clinical symptoms (fatigue, appetite, mental state, physical strength), blood glucose, blood pressure, weight, and electrolytes. Long-term excess increases risks of glucose metabolism disorders, fractures, and cardiovascular events; use the lowest effective dose whenever possible.

Criteria for Tapering and Discontinuation: For patients requiring long-term oral replacement therapy postoperatively, Serum cortisol levels were monitored at 2-week intervals during follow-up and continued until 3 months after surgery to assess recovery of the hypothalamic–pituitary–adrenal (HPA) axis and determine the presence of permanent adrenal insufficiency. If morning 8:00 a.m. cortisol > 15 μg/dL with no clinical symptoms, gradual discontinuation may be considered. If tapering is necessary, it should be conducted under endocrinology supervision with scheduled reassessments, typically following a strategy of halving the glucocorticoid dose at each follow-up. Should symptoms such as fatigue, hypotension, or hyponatremia occur after discontinuation, replacement therapy should be immediately reinstated and the patient evaluated (16). In patients with equivocal cortisol levels (3–15 μg/dL) or suspected persistent adrenal insufficiency, dynamic endocrine testing including ACTH stimulation testing was performed under endocrinology supervision. In addition to adrenal insufficiency, The ACTH stimulation test was considered normal when peak serum cortisol exceeded 18 μg/dL according to our institutional laboratory criteria. Other postoperative pituitary hormonal abnormalities, including thyroid axis dysfunction, gonadal axis dysfunction, and diabetes insipidus, were also recorded.

**Figure 3:**
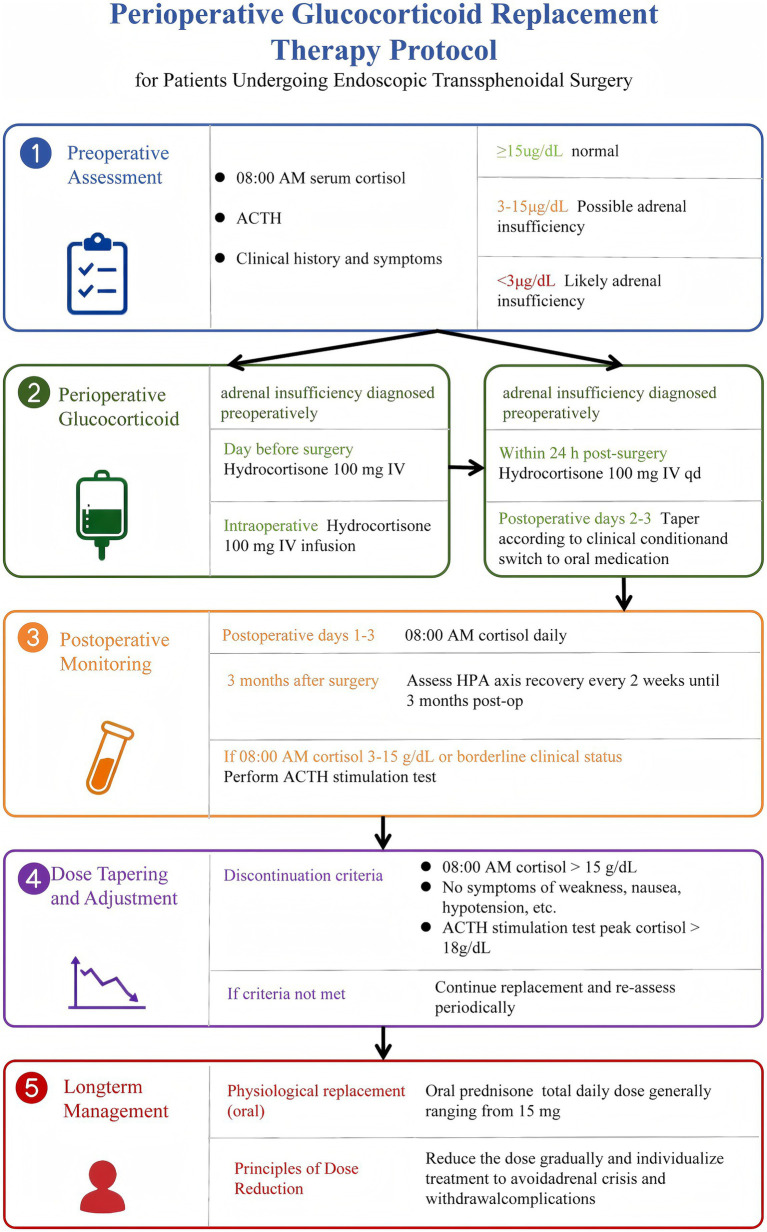
Perioperative glucocorticoid replacement and follow-up protocol.

Glucocorticoid doses were standardized to hydrocortisone-equivalent milligrams, using the following conversion ratios: hydrocortisone: cortisone acetate: prednisone: prednisolone: methylprednisolone: triamcinolone: betamethasone: dexamethasone: clobetasol = 25:20:5:5:4:4:0.8:0.75:0.5 (16–18). The body surface area (BSA) was calculated using the formula: BSA (m^2^) = 0.20247 × height (m)^(0.725) × weight (kg)^(0.425) (19). The cumulative glucocorticoid dose was calculated as: single dose × frequency × duration of therapy (20). To account for individual patient variability, we introduced the concept of glucocorticoid intensity, defined as: glucocorticoid intensity = cumulative glucocorticoid dose/BSA. All patients were followed regularly by two experienced endocrinologists, with glucocorticoid therapy adjusted according to endocrine evaluations until adrenal function recovered (Figure 3).

### Pathological study

Immunohistochemical and hematoxylin–eosin (H&E) staining were performed for pituitary hormones (FSH, LH, ACTH, TSH, PRL, GH) and transcription factors (PIT-1, TPIT, SF-1). The definition of NFPA followed the 2022 WHO classification of pituitary tumors (21). All pathological slides were reviewed by a senior pathologist (H. C.) in the Department of Pathology.

### Surgical technique

**Figure 4:**
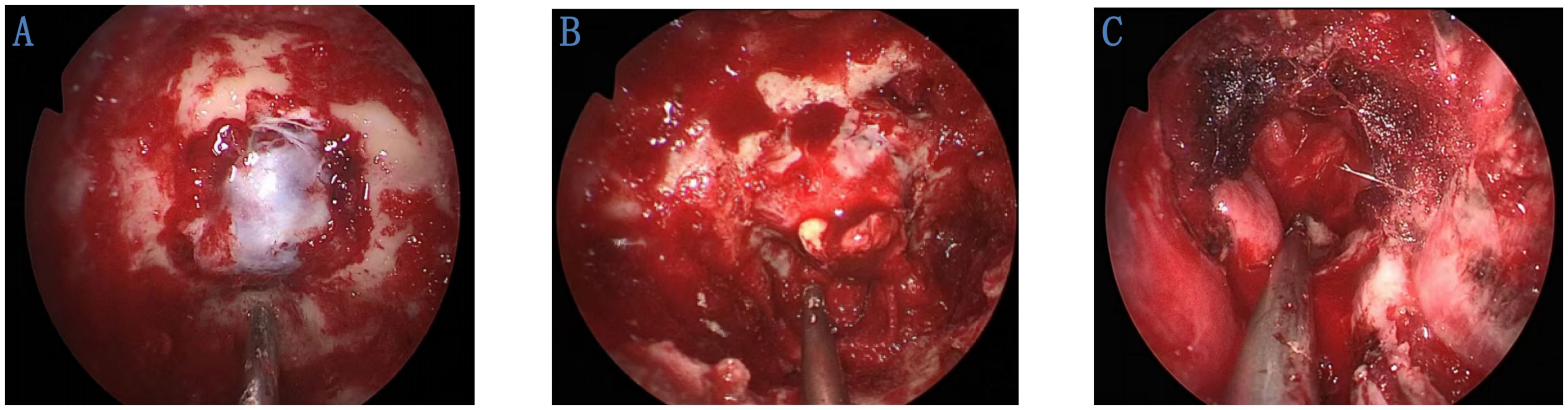
Intraoperative image of pituitary adenoma resection via transsphenoidal endoscopy. **(A)** Exposure of the sellar dura after sphenoidotomy and sellar floor opening. **(B)** Endoscopic removal of the pituitary adenoma. **(C)** Reconstruction of the sellar floor using a vascularized nasoseptal flap.

All endoscopic transsphenoidal pituitary adenoma surgeries (eTSS) were performed by two senior neurosurgeons using the binasal four-hand technique. Operative records for all 116 patients were retrospectively reviewed for detailed procedural data. The primary perioperative outcomes assessed included intraoperative blood loss and operative duration (Figure 4).

### Statistical analysis

Statistical analyses were conducted using SPSS version 25.0 (IBM Corp.). Continuous variables are expressed as mean ± SD for normally distributed data or median (interquartile range) for non-normally distributed data, while categorical variables are presented as counts and percentages. Comparisons between groups employed Student’s *t*-test for continuous variables and the *χ*^2^ test for categorical variables. Bivariate correlations were analyzed using linear regression, with statistical significance set at *p* < 0.05.

For multivariate analysis, variables with *p* < 0.05 in univariate analysis were entered into a multivariate linear regression model to identify independent predictors of glucocorticoid intensity, and create dummy variables for categorical variables. The standardized regression coefficient (β), 95% confidence interval (CI), and *p*-value were reported, and multicollinearity was assessed via the variance inflation factor (VIF > 5 indicating collinearity). Model stability was evaluated using 1,000 bootstrap resamples, with root mean square error (RMSE) and mean absolute error (MAE) reported with 95% CI (22, 23). The model was considered robust if the difference between bootstrap means and original estimates was <5% and the CI range was narrow. All tests were two-tailed with *α* = 0.05, and *p*-values were reported to three decimal places.

## Results

### Baseline characteristics of patients

A total of 116 patients who underwent pituitary tumor surgery were included in this study, comprising 58 males (50%) and 58 females (50%), with an equal gender distribution. The mean age was 51.52 ± 12.11 years, and the mean BMI was 24.27 ± 3.54 kg/m^2^. Gross total resection (GTR) was achieved in 78 patients (67.2%), whereas 38 patients (32.8%) underwent non-gross total resection (non-GTR). Mean tumor volume was 8,202.81 ± 5,282.79 mm^3^, and mean suprasellar extension was 11.46 ± 6.38 mm. The mean degree of pituitary stalk stretch was 14.79 ± 6.81 mm, and the mean morning cortisol level was 8.80 ± 6.04 μg/dL. The average intraoperative blood loss was 268.53 ± 241.85 mL, and the mean surgical duration was 266.95 ± 80.24 min. Permanent diabetes insipidus occurred in 41 patients (35.34%) postoperatively. The mean postoperative glucocorticoid intensity was 1,755.18 ± 1,092.45 mg/m^2^ (Table 1).

**Table 1:** Clinical characteristics of the patients included in this cohort.

Variable	Value (*n* = 116)	HPA axis function 1 year after surgery		
		Transient AI (*n* = 98)	Permanent AI (*n* = 18)	*P*-value
Sex				0.305
Male	58 (50%)	47 (48.0%)	11 (61.1%)	
Female	58 (50%)	51 (52.0%)	7 (38.9%)	
Age	51.5 ± 12.1	51.9 ± 11.5	49.4 ± 15.1	0.430
BMI (kg/m^2^)	24.2 ± 3.5	24.3 ± 3.5	23.9 ± 3.6	0.625
Tumor volume (mm^3^)	8021.8 ± 5379.8	6956.5 ± 4069.3	13876.1 ± 7638.6	**<0.001**
Super sellar length (mm)	11.2 ± 6.4	9.9 ± 5.7	18.3 ± 5.6	**<0.001**
Knosp class				0.380
0 ~ 2	75 (64.7%)	65 (66.3%)	10 (55.6%)	
3 ~ 4	41 (35.3%)	33 (33.7%)	8 (44.4%)	
SIR	1.8 ± 0.2	1.8 ± 0.3	1.8 ± 0.2	0.751
Stalk types				**0.023**
Stalk types 1	20 (17.2%)	19 (19.4%)	1 (5.6%)	
Stalk types 2	57 (49.1%)	51 (52.0%)	6 (33.3%)	
Stalk types 3	39 (33.6%)	28 (28.6%)	11 (61.1%)	
Optic nerve types				**0.038**
Optic nerve types 1	25 (21.6%)	24 (24.5%)	1 (5.6%)	
Optic nerve types 2	54 (46.7%)	47 (48.0%)	7 (38.9%)	
Optic nerve types 3	37 (31.8%)	27 (27.6%)	8 (44.4%)	
Pituitary stalk stretch (mm)	14.8 ± 6.8	13.3 ± 5.9	22.7 ± 5.9	**<0.001**
GTR vs. non-GTR				0.216
GTR	78 (67.2%)	68 (69.4%)	10 (55.6%)	
Non-GTR	38 (32.8%)	30 (32.6%)	8 (44.4%)	
Bleeding amount (mL)	268.5 ± 241.9	245.4 ± 234.2	394.4 ± 250.8	**0.016**
Surgery min	266.9 ± 80.2	263.8 ± 76.6	283.8 ± 97.9	0.335
PRL (ng/mL)	20.6 ± 23.9	18.4 ± 21.3	32.5 ± 32.9	**0.021**
Cortisol 8 a.m. (μg/dL)	8.8 ± 6.0	9.2 ± 5.7	6.7 ± 7.6	**<0.001**
Glucocorticoid intensity (mg/m^2^)	1755.2 ± 1092.5	1405.5 ± 685.7	3658.9 ± 920.8	**<0.001**
Permanent DI				0.732
Yes	41 (35.3%)	34 (34.7%)	7 (38.9%)	
No	75 (64.7%)	64 (65.3%)	11 (61.1%)	

AI, adrenal insufficiency; BMI, body mass index; SIR, signal intensity ratio; PRL, prolactin; DI, diabetes insipidus; GRT, gross total resection; non-GTR, non-gross total resection.

When comparing patients with transient adrenal insufficiency (AI; *n* = 98) and those with permanent AI (*n* = 18), there were no statistically significant differences in sex distribution (male 47 [40.5%] vs. 11 [9.5%], female 51 [43.9%] vs. 7 [6.0%]; *p* = 0.305), age (51.9 ± 11.5 vs. 49.4 ± 15.1 years; *p* = 0.430), or BMI (24.3 ± 3.5 vs. 23.9 ± 3.6 kg/m^2^; *p* = 0.625). The extent of cavernous sinus invasion, as reflected by Knosp grade 0–2 versus 3–4, was also comparable between the two groups (*p* = 0.380). Similarly, no significant between-group differences were observed in the signal intensity ratio on preoperative MRI (1.8 ± 0.3 vs. 1.8 ± 0.2; *p* = 0.751), extent of resection (gross total resection: 68 [69.4%] vs. 10 [55.6%]; *p* = 0.216), operative duration (263.8 ± 76.6 vs. 283.8 ± 97.9 min; *p* = 0.335), or the incidence of permanent diabetes insipidus (34 [29.3%] vs. 7 [6.0%]; *p* = 0.732).

In contrast, several radiological and perioperative parameters differed significantly between patients with transient and permanent AI. Those who developed permanent AI had substantially larger tumor volumes (13876.1 ± 7638.6 vs. 6956.5 ± 4069.3 mm^3^; *p* < 0.001) and greater suprasellar extension (18.3 ± 5.6 vs. 9.9 ± 5.7 mm; p < 0.001). The distribution of pituitary stalk types also differed significantly (*p* = 0.023): stalk type 3 was more frequent in the permanent AI group (11/18, 61.1%) than in the transient AI group (28/98, 28.6%), whereas stalk types 1–2 predominated in the transient AI group. Likewise, the distribution of optic nerve types was significantly different between groups (*p* = 0.038), with optic nerve type 3 deformation more common in patients with permanent AI (10/18, 55.6%) than in those with transient AI (27/98, 27.6%). Consistent with these more severe anatomical distortions, pituitary stalk stretch was markedly greater in the permanent AI group (22.7 ± 5.9 vs. 13.3 ± 5.9 mm; *p* < 0.001), and intraoperative blood loss was higher (394.4 ± 250.8 vs. 245.4 ± 234.2 mL; *p* = 0.016).

**Figure 5:**
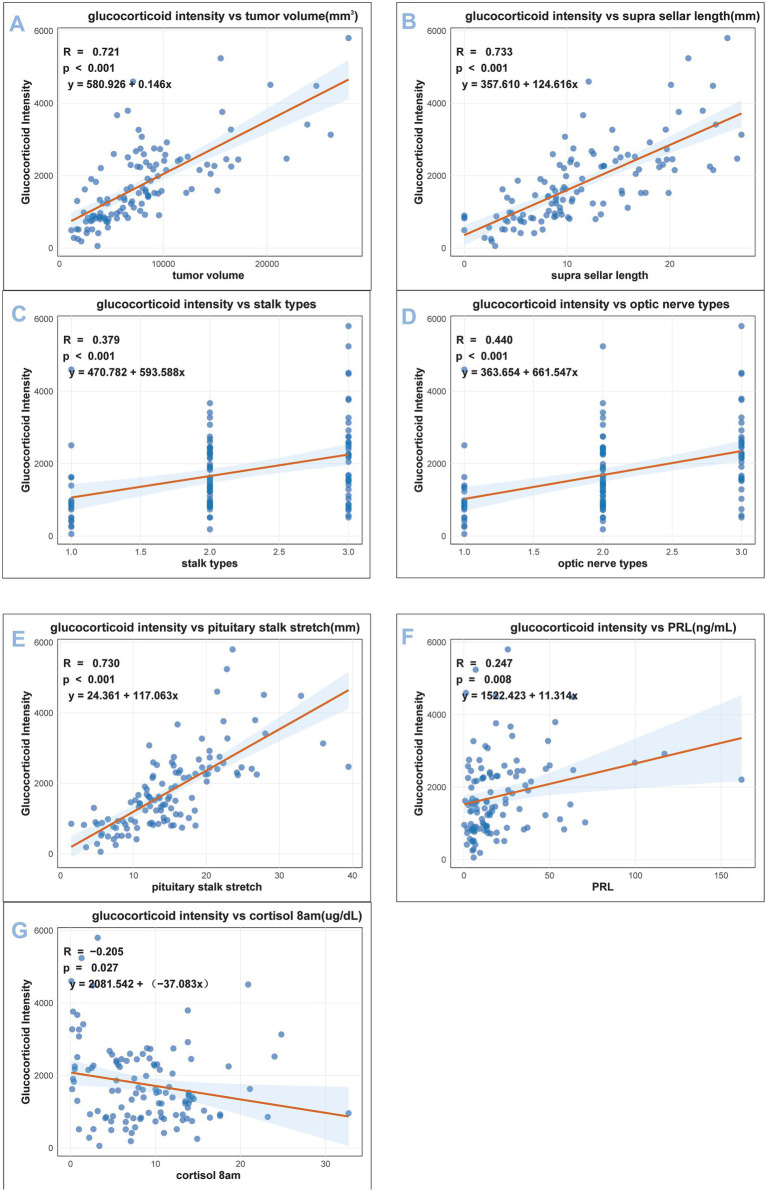
Results of univariate analysis of the relationship between clinical characteristics and postoperative glucocorticoid dosage intensity. **(A)** Glucocorticoid Intensity vs. Tumor Volume, **(B)** Glucocorticoid Intensity vs. Suprasellar Length, **(C)** Glucocorticoid Intensity vs. Stalk Types, **(D)** Glucocorticoid Intensity vs. Optic Nerve Types, **(E)** Glucocorticoid Intensity vs. Pituitary Stalk Stretch, **(F)** Glucocorticoid Intensity vs. PRL, **(G)** Glucocorticoid Intensity vs. 8 AM Cortisol.

Regarding endocrine parameters, preoperative prolactin levels were significantly higher in patients with permanent AI than in those with transient AI (32.5 ± 32.9 vs. 18.4 ± 21.3 ng/mL; *p* = 0.021), whereas 8:00 a.m. serum cortisol concentrations were significantly lower (6.7 ± 7.6 vs. 9.2 ± 5.7 μg/dL; *p* < 0.001). During follow-up, patients who developed permanent AI required substantially higher cumulative glucocorticoid replacement intensity (3658.9 ± 920.8 vs. 1405.5 ± 685.7 mg/m^2^; *p* < 0.001) than those with transient AI (Figure 5).

### Radiographic characteristics

**Figure 6:**
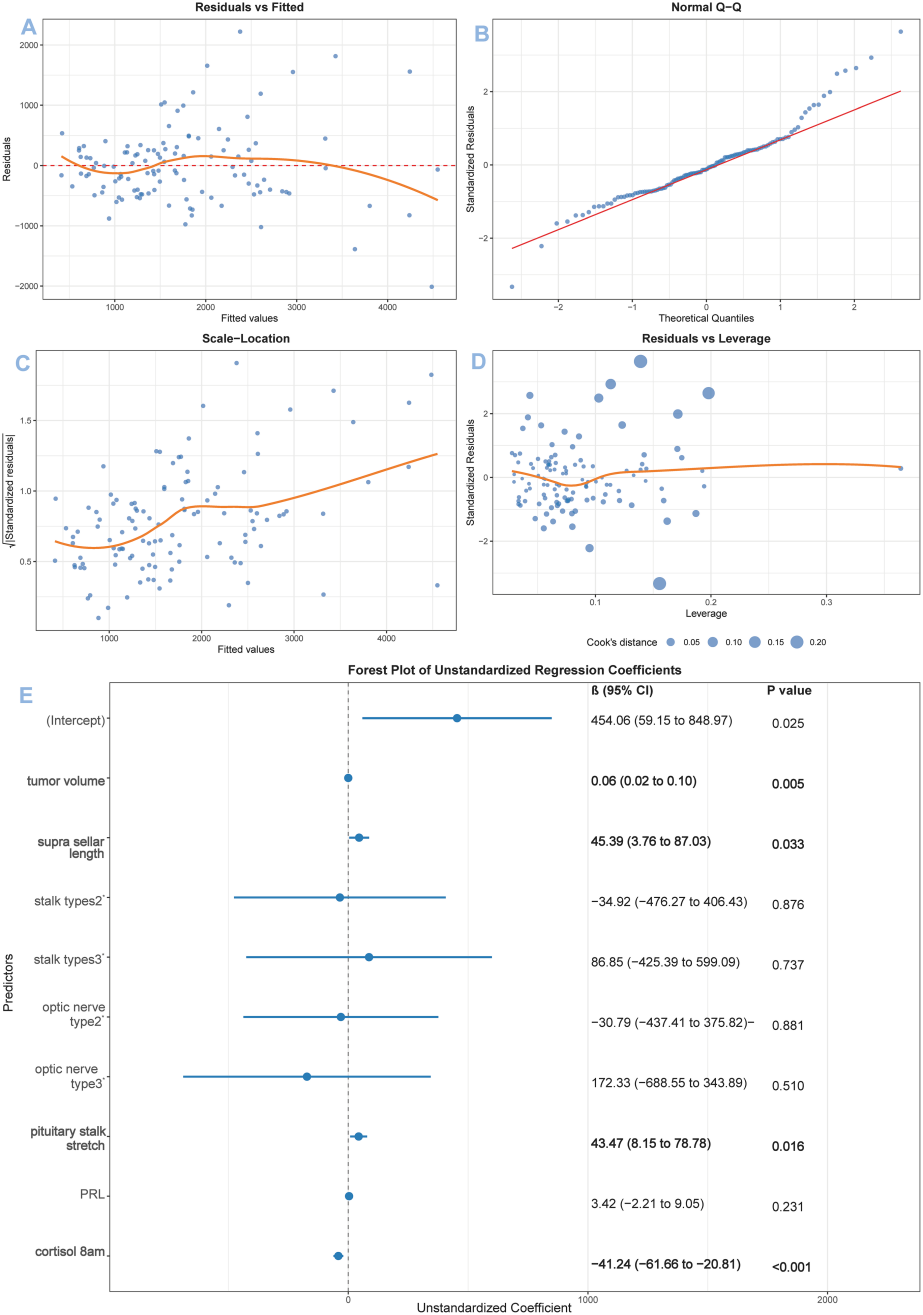
Multivariate regression analysis. **(A)** (Residuals vs. Fitted): Residuals are randomly distributed around the horizontal zero line, indicating no obvious linear relationship between residuals and fitted values, supporting the model’s linearity assumption. **(B)** (Normal Q–Q): Standardized residuals approximate the theoretical quantiles of a normal distribution, confirming the model’s normality assumption. **(C)** (Scale–Location): The spread of |standardized residuals| is uniform across fitted values, suggesting homoscedasticity (constant variance of residuals) is satisfied. **(D)** (Residuals vs. Leverage): No outliers with high leverage (leverage < 0.3) or excessive influence (Cook’s distance < 0.20) are observed, indicating the model is robust to individual data points. **(E)** Forest Plot of Unstandardized Regression Coefficients, The forest plot presents the unstandardized regression coefficients (β), 95% confidence intervals (CI), and *p*-values of predictors for glucocorticoid intensity. Asterisk represents a dummy variable set for the analysis.

Univariate regression analysis demonstrated significant positive correlations between glucocorticoid intensity and tumor volume (*β* = 0.700, 95% CI: 0.117–0.172, *p* < 0.001), suprasellar extension (*β* = 0.727, 95% CI: 102.700–146.304, *p* < 0.001), and pituitary stalk stretch (*β* = 0.730, 95% CI: 96.703–137.424, *p* < 0.001) (Figure 5). Multivariate linear regression models showed that the following factors independently influenced glucocorticoid dosage intensity: Tumor volume: Each increase of 1,000 mm^3^ was associated with an increase of 51 mg/m^2^ in dosage intensity (*β* = 0.051, 95% CI: 0.010–0.092, *p* = 0.016). Suprasellar extension: Each increase of 1 mm was associated with an increase of 45.3 mg/m^2^ in dosage intensity (*β* = 45.298, 95% CI: 4.583–86.013, *p* = 0.030). Pituitary stalk length: For every 1 mm increase, drug intensity increased by 51.1 mg/m^2^ (β = 51.146, 95% CI: 15.141–87.151, *p* = 0.006). Pituitary stalk classification (*β* = 0.370, *p* < 0.001) and optic nerve classification (*β* = 0.440, *p* < 0.001) were also statistically significant in univariate analysis (Table 2 and Figure 6).

**Table 2:** Univariate analysis and multivariate analysis.

Variable	Univariate analysis			Multivariate analysis		
	*β*	95%CI	*p*-value	*β*	95%CI	*p*-value
Tumor volume (mm^3^)	0.15	0.12 to 0.17	**<0.001**	0.06	0.02 to 0.105	**0.005**
Supra sellar length (mm)	124.62	103.18 to 146.05	**<0.001**	45.40	3.76 to 87.03	**0.033**
Stalk types	593.59	324.50 to 862.67	**<0.001**			
Stalk types 2^†^				−34.92	−476.27 to 406.43	0.876
Stalk types 3^†^				86.85	−425.39 to 599.09	0.737
Optic nerve type	661.55	411.16 to 911.93	**<0.001**			
Optic nerve type 2^†^				−30.79	−437.41 to 375.82	0.357
Optic nerve type 3^†^				−172.33	−688.55 to 343.89	0.253
Pituitary stalk stretch (mm)	117.06	96.70 to 137.42	**<0.001**	43.47	8.15 to 78.78	**0.016**
PRL (ng/mL)	11.31	3.08 to 19.55	**0.008**	3.42	−2.21 to 9.05	0.231
Cortisol 8 am (μg/dL)	−37.08	−69.93 to −4.24	**0.027**	−41.24	−61.66 to 20.81	**<0.001**
Age	−15.64	−32.13 to 0.85	0.063			
Knosp class	335.95	−81.59 to 753.49	0.114			
Permanent DI	−241.96	−661.72 to 177.83	0.249			
Sex	−202.38	−604.25 to 199.50	0.321			
Bleeding amount (mL)	0.38	−0.46 to 1.21	0.373			
Surgery min	1.08	−1.44 to 3.60	0.399			
SIR	−249.02	−1041.60 to 543.56	0.535			
GTR vs. non-GTR	280.01	−146.842 to 706.861	0.196			
BMI (kg/m^2^)	−15.44	−72.63 to 41.75	0.594			

PRL, prolactin; SIR, signal intensity ratio; DI, diabetes insipidus; BMI, body mass index; β, standardized beta coefficient; CI, confidence interval; † represents a dummy variable set for the analysis; GRT Gross Total Resection; non-GTR non-gross total resection.

### Hormone levels

Univariate analysis revealed a negative correlation between morning cortisol levels and glucocorticoid intensity (*β* = 0.027, 95% CI: −69.928 to −4.239, *p* = 0.027). In multivariate analysis, this association remained significant, with each 1 μg/dL decrease in morning cortisol associated with a 42.3 mg/m^2^ increase in glucocorticoid intensity (*β* = −42.291, 95% CI: −62.567 to −22.015, *p* < 0.001) (Table 2).

### Multivariate regression analysis

Final regression equation: Treatment intensity = 434.135 + 0.051 × tumor volume (mm^3^) + 45.298 × suprasellar length (mm) + 51.146 × pituitary stalk stretch (mm) – 42.291 × morning cortisol (μg/dL). The variance inflation factor (VIF) ranged from 1.002 to 4.508, indicating no significant multicollinearity issues.

### Model performance evaluation

Bootstrap resampling (1,000 times) demonstrated good model stability: Root Mean Square Error (RMSE): 656.04 (95% CI: 637.50–690.223) Mean Absolute Error (MAE): 485.84 (95% CI: 467.47–517.07), with Bootstrap confidence intervals for all predictor variables consistent with the original model, validating the model’s robustness. Bootstrap confidence intervals for all predictors were consistent with those from the original model, supporting robustness. The bootstrap mean estimates were closely aligned with the original coefficients (maximum difference < 5%), pituitary stalk stretch 47.445 (original) vs. 49.766 (bootstrap); cortisol −41.766 (original) vs. −41.934 (bootstrap) (Table 3).

**Table 3:** Model performance evaluation.

Term	Original estimate	Bootstrap mean	95% CI
Rmse	634.83	656.04	637.50 to 690.23
Mae	473.54	485.84	467.46 to 517.07
Tumor volume (mm^3^)	0.06	0.06	0.002 to 0.113
Aupra sellar length (mm)	43.30	43.44	9.98 to 84.41
Pituitary stalk stretch (mm)	47.44	49.77	5.31 to 94.59
Cortisol 8 am (μg/dL)	−41.77	−41.93	−65.74 to −18.95

RMSE, Root Mean Square Error; MAE, mean absolute error.

## Discussion

This study identifies independent predictors of postoperative glucocorticoid therapy intensity in patients with NFPAs following eTSS. Tumor volume, suprasellar extension, pituitary stalk stretch, and preoperative morning cortisol levels were found to significantly influence glucocorticoid requirements. Collectively, these variables account for 72.1% of the variance in glucocorticoid intensity, underscoring their potential as key clinical indicators. The predictive model developed from these parameters provides an evidence-based framework for optimizing glucocorticoid replacement therapy, which may assist clinicians in tailoring individualized treatment plans for patients.

Our multivariate analysis revealed a significant positive correlation between tumor volume and glucocorticoid intensity. For every 1,000 mm^3^ increase in tumor volume, glucocorticoid intensity increased by 51 mg/m^2^ (*β* = 0.051, *p* = 0.016). This finding aligns with prior studies that suggest larger NFPA volumes are associated with higher rates and greater severity of postoperative pituitary dysfunction, including adrenal insufficiency (24–27). The underlying mechanism may involve larger tumors exerting more substantial mechanical compression on the normal pituitary gland and surrounding structures (including the pituitary stalk and portal vein), potentially leading to more extensive ischemic injury or impaired vascular supply. Such compression may limit the extent of safe resection and affect the perfusion of the residual gland post-resection. Moreover, for larger tumors, intraoperative manipulation and glandular trauma are likely to be more severe.

Suprasellar extension was also identified as an independent predictive factor. For every 1 mm increase in extension, glucocorticoid intensity increased by 45.3 mg/m^2^ (*β* = 45.298, *p* = 0.030). Previous studies have suggested that tumor extension beyond the sella turcica is associated with a heightened risk of postoperative hypopituitarism (25). Possible mechanisms include direct compression of the pituitary stalk and its hypothalamic–pituitary portal blood supply by the tumor’s upward extension. This compression may disrupt critical vascular connections and axonal transport essential for hypothalamic control of pituitary function, particularly the hypothalamic–pituitary–adrenal (HPA) axis. Additionally, the resection of tumors with significant suprasellar extension may require more extensive manipulation near the stalk and gland, increasing the risk of direct injury.

The extent of pituitary stalk extension emerged as a strong independent predictor. For each 1-mm increase in extension, glucocorticoid intensity increased by 51.1 mg/m^2^ (*β* = 51.146, *p* = 0.006). Other studies have found that while stalk displacement is associated with postoperative diabetes insipidus, its specific relationship with HPA axis recovery and glucocorticoid demand remains unclear. This suggests a slower recovery of adrenal function postoperatively. The degree of pituitary stalk displacement may also reflect a tendency for the tumor to grow vertically preoperatively, leading to a greater degree of pituitary descent postoperatively (28–31). Thus, the extent of pituitary stalk stretching may be linked to the intensity of postoperative glucocorticoid therapy. This could be explained by the tumor compression theory, with additional unidentified mechanisms, similar to the development of postoperative diabetes insipidus, warranting further histological investigation. Significant stretching of the pituitary stalk may indicate twisting and potential damage to the hypothalamic–pituitary portal venous system. Such vascular damage may hinder the transport of hypothalamic corticotropin-releasing hormone (CRH) to the pituitary adrenocorticotropic hormone (ACTH), obstructing ACTH secretion and subsequently affecting cortisol secretion. Furthermore, stretching may reflect a tumor growth pattern preoperatively more likely to lead to postoperative pituitary insufficiency or instability, further impairing recovery.

Lower preoperative morning serum cortisol levels were found to be a strong predictor of higher postoperative glucocorticoid intensity. Each 1 μg/dL decrease in preoperative cortisol was associated with a 42.3 mg/m^2^ increase in glucocorticoid intensity (*β* = −42.291, *p* < 0.001). Preoperative adrenal function, as a baseline reserve of the HPA axis, is a well-established indicator of postoperative adrenal recovery. Lower preoperative cortisol levels are likely due to prolonged tumor compression of the gland or its stalk, resulting in preexisting subclinical or overt adrenal insufficiency. Patients with reduced preoperative cortisol reserve have less functional ACTH tissue, rendering them more vulnerable to additional surgical injury and necessitating higher and potentially longer-term glucocorticoid replacement postoperatively to prevent adrenal crisis during HPA axis recovery.

Although the Knosp grading system remains the standard for assessing cavernous sinus invasion, its predictive utility for hypothalamic–pituitary–adrenal (HPA) axis function may be inferior to imaging indicators that more directly measure compression of the stalk and suprasellar structures. In univariate analysis, elevated prolactin (PRL) levels were positively associated with glucocorticoid intensity; this relationship, however, was obscured after adjustment for imaging parameters and cortisol levels, possibly because PRL elevation reflects the tumor’s functional effects (the “stalk effect”) rather than direct HPA axis injury. We did not observe a significant association between stalk compression and postoperative glucocorticoid demand in our study, which may be attributable to limited sample size or methodological differences. Some studies have found that tumor consistency is associated with postoperative hypopituitarism; in contrast, we did not find such an association (32). This difference may reflect our reliance on MRI signal characteristics as a surrogate for tumor consistency-a method whose accuracy in predicting adenoma consistency preoperatively remains controversial.

### Strengths and limitations

The primary innovation of this study lies in the development of a predictive model based on a combination of clinical, radiological, and endocrinological parameters, providing a comprehensive approach to predicting glucocorticoid needs in NFPA patients. However, the study is limited by its retrospective design and single-center setting, which may restrict the generalizability of the findings. Further validation in larger, multicenter cohorts is needed to strengthen the model’s applicability. In addition, the extent of tumor resection and intraoperative identification of normal pituitary gland tissue may also influence postoperative adrenal function. Other intraoperative variables, including extracapsular dissection technique and degree of gland preservation, were not consistently documented in a standardized manner because of the retrospective study design. Therefore, these factors could not be reliably quantified for further statistical analysis. Future prospective studies incorporating standardized intraoperative assessments may help further clarify the relationship between surgical strategy and postoperative endocrine recovery. And future studies incorporating intraoperative imaging or fluorescence-guided techniques may further clarify the relationship between surgical strategy and postoperative recovery of the HPA axis.

## Conclusion

Multivariate regression analysis identified tumor volume, suprasellar extension, pituitary stalk stretch, and preoperative 8 a.m. morning cortisol levels as independent predictors of postoperative glucocorticoid therapy intensity in patients with pituitary tumors (all *p* < 0.05). The proposed model may serve as an evidence-based instrument for optimizing postoperative management, potentially mitigating the risk of adrenal insufficiency while minimizing the likelihood of overtreatment.

## Data Availability

The raw data supporting the conclusions of this article will be made available by the authors, without undue reservation.
